# Environmental stress gradients regulate the relative importance of predator density‐ and trait‐mediated indirect effects in oyster reef communities

**DOI:** 10.1002/ece3.7082

**Published:** 2020-12-30

**Authors:** Jessica L. Pruett, Marc J. Weissburg

**Affiliations:** ^1^ School of Biological Sciences Georgia Institute of Technology Atlanta GA USA

**Keywords:** chemical cue, density‐mediated indirect effect, environmental gradients, predation risk, trait‐mediated indirect effect, trophic cascade

## Abstract

Predators affect community structure by influencing prey density and traits, but the importance of these effects often is difficult to predict. We measured the strength of blue crab predator effects on mud crab prey consumption of juvenile oysters across a flow gradient that inflicts both physical and sensory stress to determine how the relative importance of top predator density‐mediated indirect effects (DMIEs) and trait‐mediated indirect effects (TMIEs) change within systems. Overall, TMIEs dominated in relatively benign flow conditions where blue crab predator cues increased oyster survivorship by reducing mud crab–oyster consumption. Blue crab DMIEs became more important in high sensory stress conditions, which impaired mud crab perception of blue crab chemical cues. At high physical stress, the environment benefitted oyster survival by physically constraining mud crabs. Thus, factors that structure communities may be predicted based on an understanding of how physical and sensory performances change across environmental stress gradients.

## Graphical abstract

We examined the relative importance of predator blue crab effects on intermediate prey mud crab consumption of basal resource oysters across a flow gradient which imposes physical and sensory stress. We found that the environment simultaneously imposes physical and sensory stressors that modify the relative strengths of predator density‐ and trait‐mediated indirect effects through distinct processes.

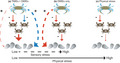

## 1 INTRODUCTION

Predation is fundamentally important in the structure and function of ecosystems (Ripple et al., 2014). Top predators in a tritrophic food chain can indirectly impact lower trophic levels by reducing intermediate prey density through consumption (i.e., consumptive effects). Density‐mediated indirect effects (DMIEs) affecting the prey of these intermediate consumers have been well‐documented to influence diverse ecological communities (Carpenter et al., 1987; Estes & Palmisano, 1974; Power et al., 1985; Shurin et al., 2010; Terborgh et al., 2006). In addition to consumptive effects, the presence of predators can induce behavioral, morphological, or life‐history changes in prey traits, which has indirect effects on lower trophic levels (predation‐risk effects sensu Peacor et al., 2020). These trait‐mediated indirect effects (TMIEs) can be as strong or stronger than DMIEs (Preisser et al., 2005; Werner & Peacor, 2003). However, recent long‐term field studies investigating the relative importance of DMIEs versus TMIEs reveal contrasting conclusions about the strength of TMIEs in natural systems (Kimbro et al., 2017; Rinehart et al., 2017; Wada et al., 2017).

Environmental conditions are known to modulate the relative importance of consumptive effects (Bertness et al., 2002; Leonard et al., 1998; Menge, 1978; Preisser & Strong, 2004; Shears et al., 2008). The environmental stress model predicts that prey are released from predation pressure at high physical stress levels due to reduced predator motility and foraging abilities in harsh environments that impose strong mechanical forces (Menge & Sutherland, 1987). For example, wind is a physical stressor in terrestrial systems that hinders predatory ladybeetle foraging on aphids, which results in increased aphid abundances on soybean plants (Barton, 2014). However, the environmental stress model does not consider that within physically mild conditions some environmental forces can reduce the ability of prey to detect predators (i.e., sensory stress), which may influence the magnitude of consumptive and predation‐risk effects.

Prey detect and assess predation threats by using surrounding smells, sounds, and sights associated with predation or predators (Munoz & Blumstein, 2012; Weissburg et al., 2014). Yet, the physical environment alters prey sensory capabilities (Jacobs et al., 2008; Large et al., 2011; Robinson et al., 2007) and so may modify predation‐risk effects. Fish prey species respond to visual predator cues in clear, but not turbid, water due to impaired visual perception (Becker & Gabor, 2012; Hartman & Abrahams, 2000). Anthropogenic sensory stressors, such as noise and light pollution, affect prey ability to detect predators as well (Barber et al., 2010; Halfwerk & Slabbekoorn, 2015). Several species of ground foraging birds reduce the distance at which they respond to an approaching predator stimulus in more noise polluted areas (Petrelli et al., 2017). Many other environmental gradients affect various sensory modalities at levels that are not necessarily stressful physically (Weissburg et al., 2014), but we lack investigations that explore the effect of sensory stress on TMIEs. Some environmental features, such as fluid flow, can both physically constrain animal locomotion and diminish sensory perception (Cherry & Barton, 2017; Weissburg et al., 2003), which further complicates the relationship of predator controls across environmental gradients.

Drawing from consumer stress models and prey stress models (Menge & Olson, 1990), the “sensory stress model” suggests the relative importance of consumptive versus predation‐risk effects is dependent on whether predator or prey sensory perception declines more rapidly with increasing sensory stress (Smee et al., 2010; Weissburg et al., 2014). If prey are more affected by sensory stress than predators, predation‐risk effects will decrease as sensory stress increases and consumptive effects will increase until the point at which predator sensory detection is comprised. Clams reduce activity in response to blue crab cues in low sensory stress flow conditions which decreases blue crab predation rates, yet at intermediate stress levels clams are unable to detect and respond to predation threat and blue crab predation rates increase (Smee et al., 2010). However, blue crab consumptive effects decrease in high sensory stress flow environments because their ability to locate clams is compromised (Smee et al., 2010). Yet, if predators are more affected by sensory stress compared to prey, consumptive effects will decrease in strength as sensory stress increases and predation‐risk effects will remain important until a maximum at which prey detection of predators begins to decline. Piscivorous fish foraging rates decline more rapidly than planktivorous fish with increasing turbidity due to differences in distance at which they must visually detect their prey (De Robertis et al., 2003). However, planktivorous fish foraging rates only increase gradually with turbidity due to piscivorous fish predation‐risk effects decreasing activity at turbidity levels at which consumptive effects are limited (Pangle et al., 2012). This framework has not been experimentally tested in a tritrophic system to examine how DMIEs and TMIEs change across an environmental stress gradient.

We used a tritrophic food chain (blue crab‐mud crab–oyster) common in oyster reefs to assess the importance of environmental gradients that impose both physical and sensory stress on top‐down predator effects. Tidally driven flows vary spatially and temporally in estuarine systems (Wilson et al., 2013), which has the capacity to regulate predator controls. Blue crabs, *Callinectes sapidus*, are mobile predators found commonly in salt marsh communities that feed primarily on smaller crustaceans and bivalves (Byers et al., 2017; Laughlin, 1982; Micheli, 1997). Fluid forces restrict blue crab locomotion (Weissburg et al., 2003) and turbulence interferes with blue crab sensory ability to locate prey (Weissburg & Zimmer‐Faust, 1993), which limits blue crab top‐down effects in high flow conditions (Smee et al., 2010). Mud crabs, *Panopeus herbstii*, are small xanthid crabs that live within the interstitial spaces of oyster reefs (Meyer, 1994) and prey heavily on recently settled oysters (Bisker & Castagna, 1987; Rindone & Eggleston, 2011; Toscano & Griffen, 2012). Mud crabs are readily consumed by blue crab predators (Grabowski et al., 2008; Hill & Weissburg, 2013a) and respond to chemical cues from blue crabs by reducing foraging on juvenile oysters (Hill & Weissburg, 2013b; Weissburg et al., 2016). While both blue crabs and mud crabs are consumers of juvenile oysters, the small body size and crushing claw morphology of mud crabs make them a more efficient oyster predator within oyster reefs (Carroll et al., 2015; Hill & Weissburg, 2013a). Flow conditions affect mud crab foraging performance at high flow velocities and the distance at which mud crabs detect and respond to blue crab chemical cues decreases as turbulence increases (Pruett & Weissburg, 2018). Thus, oyster survivorship may vary along flow gradients based on the interaction of physical and sensory stressors modulating blue crab‐mud crab dynamics.

This study examined the effect of stress gradients on blue crab DMIEs and TMIEs by determining oyster survival in the presence of simulated blue crab predation, predation‐risk blue crabs, and lethal blue crabs at different flow regimes within the same estuary. These site and tidal type combinations previously demonstrated hydrodynamic effects on mud crab physical ability to consume oysters and chemosensory detection of blue crabs (Pruett & Weissburg, 2018). We predicted at low environmental stress conditions, blue crab TMIEs would dominate because mud crabs are able to detect and respond to blue crab risk cues, which will increase oyster survivorship. The outcomes in high sensory stress environments with intermediate physical stress depend on if blue crab sensory detection of mud crabs is impaired at the same turbulence levels that limit mud crab perception of blue crabs. Thus, either blue crab DMIE strength will increase in high turbulent flow or mud crabs will be released from blue crab predator controls. Lastly, at high physical stress, blue crab effects on oyster survival will not be important because hydrodynamic forcing limits mud crab movement and the environment enhances oyster survival.

## 2 METHODS

### 2.1 Animal collection and maintenance

Blue crabs and mud crabs were obtained from Wassaw Sound (Savannah, GA, USA) and associated tributaries. Blue crabs were collected using baited crab traps. Mud crabs were caught by hand during low tide from oyster reefs. A scientific collecting permit (29‐WJH‐16‐222) issued by the Georgia Department of Natural Resources approved the collections and was renewed annually. Oyster spat (10–16 mm hinge length) were purchased from local commercial hatcheries. All animals were housed in separate flow‐through seawater systems at the Skidaway Institute of Oceanography (SkIO). Blue crabs (12–16 cm carapace width (CW)) were maintained individually and fed an ad libitum diet of mud crabs beginning 48 hr prior to a field trial. Blue crabs fed mud crabs produce chemical cues with stronger deterrent effects than blue crabs fed other diets (Weissburg et al., 2016). Mud crabs were housed based on CW size classes (15–20, 20–25, 25–30 mm) to prevent cannibalism and fed an ad libitum diet of oysters every 2 days.

### 2.2 Site description

Field experiments were performed at two sites in tributaries of Wassaw Sound. Priest Landing site (PL; 31°57′41.4″ N, 81°0′45″ W) was in Wilmington River, which was downstream of the Skidaway Narrows site (SN; 31°57′16.2″ N, 81°3′41.04″ W) located in Skidaway River. Tidal range in these areas is 2–3 m, with water temperature and salinity 25–30°C and 20–28 ppt respectively, during the summer period when our experiments took place. Both sites contain mudflats bordered by *Spartina alterniflora* salt marshes.

### 2.3 Flow estimation

Flow conditions significantly vary between these sites based on previous extensive flow measurements by Wilson et al. (2013). PL is characterized by higher mean turbulent kinetic energy (TKE) and slower current speed relative to SN, which has faster mean current speed and lower TKE (Pruett & Weissburg, 2018; Wilson et al., 2013). Flow parameters are strongly related to tidal range and at each site can be predicted using the relationship between tidal range and either current speed or TKE (Pruett & Weissburg, 2018; Wilson, 2011). Regression equations calculated by Pruett and Weissburg (2018), which were obtained from flow measurements acquired by Wilson et al. (2013), were used to estimate flow conditions during our trials.

### 2.4 Field experiment

We measured the strength of blue crab predator effects on mud crab consumption of juvenile oysters across physical (i.e., current speed) and sensory (i.e., turbulence) stress gradients. Experiments were conducted on intertidal mudflats about one tidal foot below mean low water. Mud crab enclosure cages (1.25 m × 1.25 m × 0.3 m; l × w × h) consisted of PVC frames covered by 1 cm^2^ vexar mesh. Flow conditions inside the cages are within the range, and reflective of, the natural conditions documented outside the cages (Hill & Weissburg, 2013b; Wilson et al., 2013). An oyster reef was constructed in the center of the enclosure to provide a habitat for mud crabs. The oyster reef was created using a combination of four natural oyster clusters (~0.2 m dia.) and four artificial oyster clusters. Natural oyster clusters were sun bleached to remove live organisms that could provide additional cue sources or food resources but maintain the natural reef structure. Smaller artificial oyster clusters (~6 cm dia.) were created by attaching several sun‐bleached oyster shells together. Artificial oyster clusters were used to manipulate the placement of oyster spat within the cages. Four oyster spat (10–16 mm) were attached to the surface of artificial clusters using marine epoxy. Four artificial oyster clusters were interspersed within the oyster reef and an additional four artificial oyster clusters were placed 0.3 m away from the reef equidistant from each other. In total, each cage contained 32 oyster spat with 16 spat each inside and outside the reef.

Each enclosure also contained mud crabs and a blue crab predator treatment. Fifteen mud crabs (8 crabs 15–20 mm CW, 4 crabs 20–25 mm CW, and 3 crabs 25–30 mm CW), which mimicked local size distribution and density (Hill & Weissburg, 2013b), were added to the enclosure. Mud crabs were painted with bright paint markers to distinguish from possible mud crab immigrants, but no immigrating mud crabs were found in any cages. Enclosures were assigned to one of four blue crab predator treatments: no‐blue crab control, mud crab cull (consumptive effect only), predation‐risk blue crab, or lethal blue crab (consumptive & predation‐risk effects). The mud crab cull treatment simulated blue crab predation without the presence of blue crab cues and consisted of removing 5 mud crabs (3 crabs of 15–20 mm CW, 1 crab of 20–25 mm CW, 1 crab of 25–30 mm CW). The culling rate and distribution were based on preliminary experiments that measured blue crab 24‐hr predation rate on mud crabs in enclosures with the same experimental setup as the field experiment. Predation‐risk blue crabs were mobile and able to release chemical cues but chelipeds were clamped shut with heat‐shrink tubing covered by duct tape and cinched down by a cable tie to prevent blue crabs from attacking mud crabs. Lethal blue crabs were unrestrained and able to consume mud crabs as well as release chemical cues. Predation‐risk blue crabs were replaced after 24 hr to match the predation‐risk effect strength of an actively foraging blue crab (i.e., lethal blue crab) because blue crab predation‐risk effects decrease after 24 hr if not fed (Weissburg & Beauvais, 2015).

The number of surviving oysters and remaining mud crabs were counted after 48 hr in each enclosure. Each 48‐hr block had 3 replicates per treatment that were randomly placed 5 m apart. The number of replicates was constrained by the limited amount of time enclosures could be set up and taken down during low tide mudflat exposure. The distance between sites required that trials on a given date could be deployed at only one site. Trials occurred either at mean or spring tide, assigned according to the average low tide height during the 48‐hr block (Wilson et al., 2013). Mean tide low tide heights ranged between −0.07 and 0.29 m, while spring tide low tide heights were between −0.37 and −0.09 m. The average tidal range for each site and tidal type combination blocks was 2.24 and 2.25 m during mean tide for PL and SN respectively, and 2.65 m for PL and 2.60 m for SN during spring tide.

We performed 24 trial blocks from 2015 to 2018 in the months of late May through early August. One block for PL at mean tide experienced much higher water temperatures (>30°C) and air temperatures (>37°C) than the other blocks so was omitted from data analysis. One block for SN during spring tide occurred during a tropical storm and was removed from analysis as well. Occasionally (7 out of 22 blocks), there were not enough animals to perform three replicates of each blue crab treatment, so a third replicate of some treatments was omitted. One block at PL during mean tide only had two replicates of each blue crab treatment due to cages shifting during the experiment.

### 2.5 Statistical analysis

Mud crab recovery after 48 hr was analyzed using a mixed‐effects model fit by REML to assess the effect of blue crab predator treatment on mud crab densities and the effect of site and tidal type. Oyster survival was analyzed using a generalized linear mixed‐effects model with binomial error and logit link. Fixed effects for both models were site, tidal type, and blue crab predator treatment with block date as a random effect. Mixed‐effects model analysis was performed using the lme4 package (Bates et al., 2015) for R version 3.3.1 (R Core Team, 2017). The degrees of freedom and *p* values for the mixed‐effects model were calculated using Kenward‐approximations in the lmerTest package (Kuznetsova et al., 2017). Any post hoc comparisons were done using the lsmeans package (Lenth, 2016).

We calculated the effect size of blue crab predator effects on oyster survival using ratio‐based indices (Okuyama & Bolker, 2007; Trussell et al., 2006). We defined DMIE, TMIE, and total predator indirect effect (TPIE) as:

$$\text{DMIE}=\frac{\text{oyster}\;\text{survival}\;\text{with}\;\text{lethal}\;\text{blue}\;\text{crab}}{\text{oyster}\;\text{survival}\;\text{with}\;\text{predation}‐\text{risk}\;\text{blue}\;\text{crab}}‐1.$$

$$\text{TMIE}=\frac{\text{oyster}\;\text{survival}\;\text{with}\;\text{predation}‐\text{risk}\;\text{blue}\;\text{crab}}{\text{oyster}\;\text{survival}\;\text{with}\;\text{no}\;\text{blue}\;\text{crab}}‐1.$$

$$\text{TPIE}=\frac{\text{oyster}\;\text{survival}\;\text{with}\;\text{lethal}\;\text{blue}\;\text{crab}}{\text{oyster}\;\text{survival}\;\text{with}\;\text{no}\;\text{blue}\;\text{crab}}‐1.$$

**FIGURE 1:**
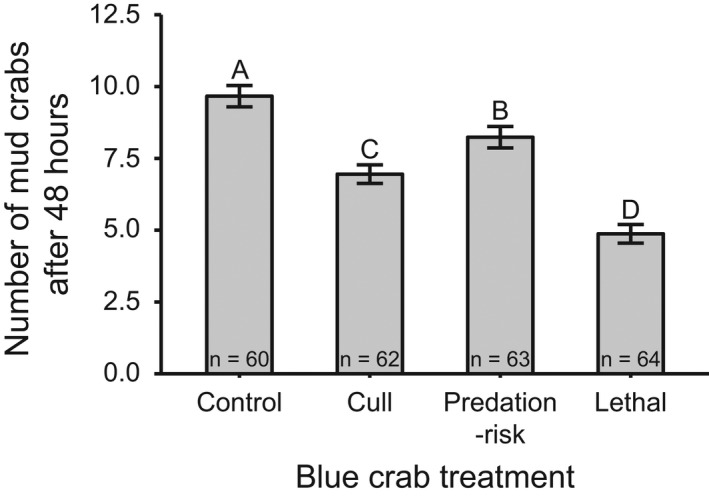
Number of mud crabs (mean ± *SE*) recovered from cages after 48 hr for each blue crab predator treatments. Different letters denote means that are significantly different based on Tukey post hoc tests (*p* < .05)

The numerator was the number of surviving oysters in a single replicate for the stated treatment and the denominator was the average number of surviving oysters of the stated treatment for a given trial block. This results in three replicate values for each effect size per trial block that were all included in the multiple regression analyses described below. We calculated DMIEs by comparing oyster survival in the lethal blue crab treatment to the predation‐risk blue crab treatment because the cull treatment did not mimic the same predation rate as the lethal treatment and may have underestimated DMIEs (see Section 3; Figure 1). Positive effect sizes indicate blue crabs benefit oyster survival.

We used multiple linear regression to analyze the relationship between blue crab predator effect size and flow properties. Flow measurements during each trial block were estimated using the predictive relationship between tidal range and flow conditions (regression equations derived by Pruett and Weissburg (2018)). Multiple linear regression analyses were performed in R, in which blue crab DMIE or TMIE size was regressed against current speed and TKE. Current speed was not a significant predictor in any of the multiple linear regressions (*p* > .05), so was dropped from the regressions. Effect sizes were log‐transformed to meet normality assumptions.

## 3 RESULTS

### 3.1 Mud crab recovery

Only blue crab treatment had a significant effect on the number of mud crabs recovered after 48 hr, with the greatest number of mud crabs recovered in the control and predation‐risk blue crab treatments and lowest in the cull and lethal blue crab treatments (Figure 1 and Table 1). The difference in mud crab final densities between the cull and lethal treatment suggests the cull treatment did not sufficiently mimic true blue crab consumption rates on mud crabs.

**TABLE 1:** Mixed‐effects model analysis of the effects of site, tidal type, and blue crab treatment on the number of mud crabs recovered after 48 hr

Source	*df* (num, den)	*F‐*value	*p*‐value
Site	1, 18.0	0.366	.553
Tidal type	1, 18.0	0.036	.852
Treatment	3, 215.3	40.2	<.001*
Site × Tidal type	1, 18.0	0.800	.383
Site × Treatment	3, 215.3	0.539	.656
Tidal type × Treatment	3, 215.3	0.587	.624
Site × Tidal type × Treatment	3, 215.3	0.771	.511

Note

Significance did not change with removal of three‐way interaction from the model.

* Significant *p* < .05.

### 3.2 Oyster survival

**FIGURE 2:**
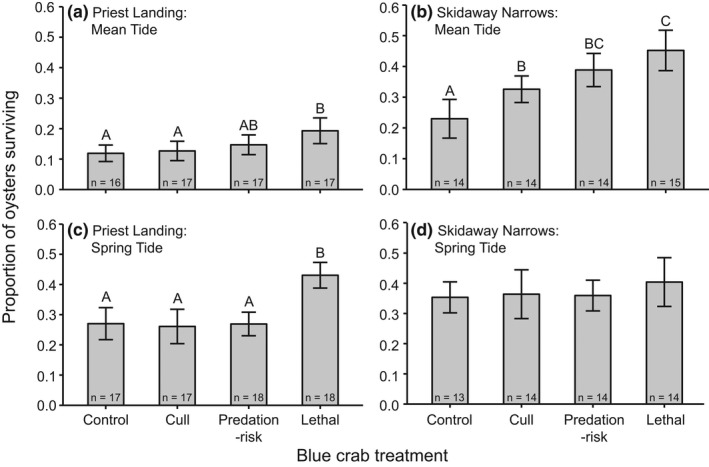
Proportion of oysters surviving (mean ± *SE*) after 48 hr in each blue crab treatment during mean tide at (a) Priest Landing (PL) and (b) Skidaway Narrows (SN) and during spring tide at (c) PL and (d) SN. Different letters denote means that are significantly different based on Tukey post hoc tests (*p* < .05) within these site and tidal type combinations

Blue crabs had significant indirect effects on oyster survival, but this was dependent on site and tidal type combination (Table 2). In general, oyster survival was lowest at the Priest Landing (PL) site during mean tide but significantly higher in the lethal blue crab treatment compared to the no‐blue crab control at this site and tidal type combination (Figure 2a). Oyster survivorship was greater in the presence of blue crab predator effects at the Skidaway Narrows (SN) site during mean tide with an ~80% increase in survivorship when predation‐risk or lethal blue crabs were present relative to the no‐blue crab control (Figure 2b). At PL during spring tide, oyster survival was highest in the presence of lethal predator blue crabs compared to the other blue crab treatments and control (Figure 2c). Oyster survival in the no‐blue crab control was relatively higher at SN during spring tide compared to the other site and tidal types and similar among all the blue crab treatments (Figure 2d).

**TABLE 2:** General linear mixed‐effects model analysis of the effects of site, tidal type, and blue crab treatment on the proportion of oysters surviving after 48 hr

Source	*df*	Chi square	*p*‐value
Site	1	7.78	.005*
Tidal type	1	3.74	.053
Treatment	3	99.6	<.001*
Site × Tidal type	1	2.24	.134
Site × Treatment	3	11.5	.009*
Tidal type × Treatment	3	6.51	.089
Site × Tidal type × Treatment	3	12.0	.007*

Note

Analysis of deviance results were fit with logistic regression analysis (general linear model with binomial error distribution and logit link).

* Significant *p* < .05.

**FIGURE 3:**
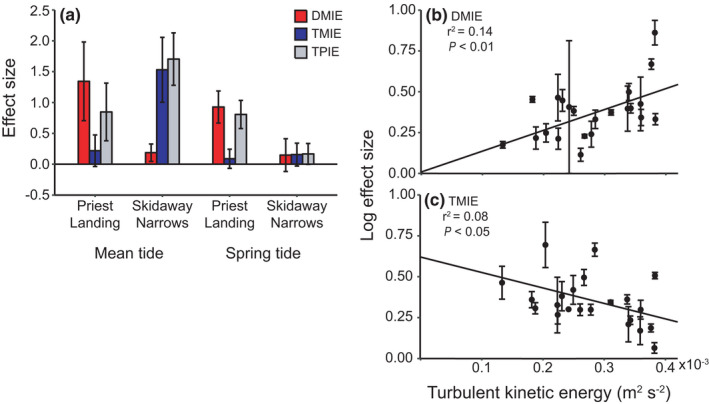
(a) Blue crab density‐mediated indirect (DMIE, red bars), trait‐mediated indirect (TMIE, blue bars), and total predator indirect (TPIE, gray bars) effect size (mean ± *SE*) on oyster survival at each site and tidal type. The log strength of (b) DMIEs and (c) TMIEs on oyster survival during each trial block (mean ± *SE*) as a function of estimated turbulent kinetic energy (TKE, m^2^/s^2^)

The effect size of blue crabs on oyster survival differed between the site and tidal type combinations, in which the relative contribution of DMIEs versus TMIEs depended on the estimated flow environment (Figure 3). DMIEs were strongest at PL (Figure 3a), which had higher turbulence levels than SN. The strength of DMIEs on oyster survival had a positive linear relationship with estimated TKE (Figure 3b; *F*_1,62_ = 11.5, *p* = .0012, *r* ^2^ = .14). TMIEs were strongest at SN during mean tide (Figure 3a), which had the lowest TKEs relative to the other site and tidal type combinations. The strength of TMIEs on oyster survival decreased with increasing estimated TKE (Figure 3c; *F*_1,61_ = 6.23, *p* = .015, *r* ^2^ = .078). Neither DMIEs nor TMIEs had appreciable effects on oyster survival at SN during spring tide, which had the highest current speeds (Figure 3a).

## 4 DISCUSSION

**FIGURE 4:**
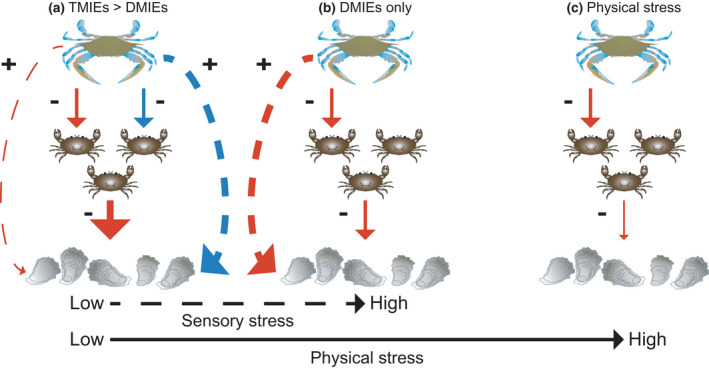
Conceptual model of how blue crab predator consumptive (red) and predation‐risk (blue) effect strengths change across a flow gradient that imposes sensory stress at high turbulence and physical stress at high current speeds. Solid arrows refer to direct blue crab effects on mud crabs and dotted lines refer to indirect blue crab effects on oysters. Arrow thickness corresponds to magnitude of effect strength. (a) At low sensory and physical stress, mud crab reactive range is large due to low sensory stress and blue crab predation‐risk effects decrease mud crab–oyster consumption which has strong positive trait‐mediated indirect effects (TMIEs) on oyster survival. (b) Mud crab sensory perception is impaired at high sensory stress and blue crab predation‐risk effects are not important. Blue crab density‐mediated indirect effects (DMIEs) increase oyster survival by reducing mud crab densities in high sensory stress and intermediate physical stress conditions. (c) High physical stress constrains mud crab foraging abilities which increases oyster survival. Blue crabs only have direct consumptive effects on mud crab survival. Blue crab and oyster images from the Integration & Application Network, University of Maryland Center for Environmental Science (https://ian.umces.edu/symbols/). Mud crab image by the author, J. Pruett

Our results suggest that the importance of blue crab DMIEs and TMIEs within an estuary changes across a flow gradient that impacts mud crab physical and sensory performance (Figure 4). Blue crab positive TMIEs on oyster survival were strong at low estimated TKEs but decreased in magnitude as sensory stress increased and the ability of mud crabs to chemically detect blue crab predators diminished. In return, blue crab DMIEs grew in importance as sensory stress increased and were highest under the maximum estimated TKEs. Oyster survival was relatively high regardless of blue crab predator treatments at the fastest estimated current speeds, which physically restricted mud crab consumption of oysters.

Predator TMIEs regulated total predator effects in low sensory stress conditions that permit prey detection of predators (Figure 4a). Blue crab TMIEs were strongest in the low estimated TKE conditions at SN during mean tide (Figure 3a), where oyster survival was higher in the predation‐risk blue crab treatment compared to the no‐blue crab control (Figure 2b). TMIEs strength decreased as turbulence increased (Figure 3c). Higher turbulent mixing changes chemical plume structures by diluting odor burst signal strength (Jackson et al., 2007; Koehl, 2006), which reduces predator and prey chemosensory abilities (Powers & Kittinger, 2002; Smee et al., 2008; Weissburg & Zimmer‐Faust, 1993). The lack of blue crab TMIEs at PL likely is a result of reduced mud crab ability to detect blue crab chemical cues at this site, which has generally higher estimated TKE than SN. This is consistent with the analysis showing TMIE strength declines as sensory stress increases (Figure 3c) and prey sensory ability to detect predators decreases. Other studies in aquatic habits show similar effects of sensory stressors. Predator chemical cues reduce whelk consumption of barnacles, but the whelk antipredator response decreases in intensity along a sublethal copper concentration gradient that inhibits whelk chemosensory abilities at higher concentrations (Kwan et al., 2015).

At high sensory stress levels predator consumptive effects were more important than predation‐risk effects due to declines in prey sensory performance (Figure 4b). Blue crab DMIEs increased with sensory stress as the importance of TMIEs declined (Figure 3b,c). Oyster survival was similar between the no‐blue crab control and predation‐risk blue crab treatment at PL during spring tide, which had the highest estimated TKE levels, but lethal blue crab predators indirectly increased oyster survival in these turbulent conditions (Figure 2c). Oyster survivorship has been shown to increase with decreasing mud crab densities in both laboratory (Hughes et al., 2012) and field studies (Kimbro et al., 2017). We did not see a positive effect on oyster survival at this site and tidal type in the cull treatment (Figure 2c), which attempted to mimic the blue crab predation rates on mud crabs in the lethal treatment, but average final mud crab density was about 30% higher in the cull treatment compared to lethal blue crab cages (Figure 1), suggesting this level of mud crab reduction was insufficient to have beneficial effects on oyster survival. Thus, the positive blue crab effect on oyster survival in the lethal blue crab treatment at PL during spring tide was a result of decreased mud crab density by blue crab predators that were still able to successfully locate mud crab prey at sensory stress levels that greatly hindered mud crab detection of blue crab predators.

Conversely, predator sensory ability to locate prey can be more affected by the same sensory stressor that degrades prey ability to detect predators. In these scenarios it is predicted that consumptive effects will decrease in strength as sensory stress increases and predation‐risk effects will remain important until the point at which prey detection of predators begins to decline (Weissburg et al., 2014). In tritrophic systems, intermediate prey will be released from top predator consumptive and predation‐risk effects at sensory stress levels that hinder top predator ability to locate intermediate prey and intermediate prey ability to detect top predators but not their ability to locate basal resources. Basal resource survivorship will be determined by how sensory stress affects intermediate prey ability to locate basal resources in these situations. For example, anthropogenic noises reduce lady beetle predation rates on aphids by potentially disrupting vibration detection of prey but does not affect aphid reduction of plant biomass, and consequently, aphids are released from top‐down lady beetle effects in the presence of noise pollution (Barton et al., 2018). Thus, estimates of the sensory performance of both predators and prey across the same gradient provide a framework to understand how sensory stress may structure communities by changing cascading predator effects.

Environmental stress models (Menge & Olson, 1990; Menge & Sutherland, 1987) predicted the outcome of interactions at high physical stress, in which blue crab predator effects were negligible because mud oyster consumption was constrained by hydrodynamic physical forcing (Figure 4c). At SN during spring tide, which is characterized by the fastest current speeds, but intermediate turbulence compared to the other site and tidal type combinations, oyster survival was relatively high regardless of predator treatment (Figure 2d) and blue crab indirect predator effects were minimal (Figure 3a). Previous results from SN during spring tide (Pruett & Weissburg, 2018) and other field studies measuring reef‐dwelling crab species foraging rates on bivalves (Leonard et al., 1998; Robinson et al., 2011) demonstrate depressed crab foraging in higher flow speed. Top predators can also be physically hampered by environmental stressors that reduce their lethal effects on intermediate prey, which may affect lower trophic levels negatively if intermediate consumers still are able to forage successfully under these stressful conditions. Sparrowhawk attack success on the shorebird redshanks decreases with wind speed due to reduced flight control in high wind conditions and an increased ability of redshanks to evade hawk attacks (Quinn & Cresswell, 2004). Thus, shorebird consumption of invertebrate prey may increase under these conditions in which their ability to evade hawk attacks and forage are not compromised (Cherry & Barton, 2017). In physically harsh contexts, sensory stress no longer regulates predator controls but how physical stress simultaneously affects predator and prey performance (i.e., consumer/prey stress models; Menge & Olson, 1990) will determine basal resource abundance.

This study suggests that the flow environment, which inflicts physical and sensory stress, can be a driving force in regulating the relative importance of top predator controls across oyster reefs within an estuary. Although we performed field experiments in only one estuary, the flow variations we see are typical (e.g., Chanson et al., 2012), and therefore expect the general results to be valid. Variations in topography and substrate may affect the specific current speeds at which different effects are seen but the transitions between sensory stress dominated regulation and physical forcing is likely to occur. The strength of trophic cascades within oyster reefs change across broad regional environmental gradients (Kimbro et al., 2014), which further supports the idea that changes in sensory and physical stressors can shift patterns of regulation. We believe our results provide a general framework that merits testing across ecosystems for predicting the relative strength of DMIIs and TMIIs in tritrophic systems in which prey sensory detection of predators declines more rapidly than their physical ability to forage along an environmental stress gradient. Odor‐mediated predator–prey interactions across a diverse array of taxa are prevalent in both aquatic and terrestrial systems that contain environmental features that impose substantial sensory stress at levels that are not physically limiting (Dicke & Grostal, 2001; Ferrari et al., 2010; Kats & Dill, 1998; Parsons et al., 2018). Like in aquatic environments, wind can physically constrain animal locomotion and alter odor plume structure to reduce chemosensory perception (Cherry & Barton, 2017; Wilson et al., 2015). Other sensory modalities used by prey to detect predators are also impaired in environments that are not physically harmful, such as visual detection in turbid waters (Chivers et al., 2013) and mechanosensory abilities in moderate turbulence (Buskey et al., 2012). Anthropogenic stressors often affect sensory processes through increased background noise, altered quality or quantity of risk cues, or disturbed sensory mechanisms before harming physical or physiological performance (Halfwerk & Slabbekoorn, 2015; Leduc et al., 2013; Lurling & Scheffer, 2007). Thus, we anticipate understanding how predator and prey sensory performances change across environmental conditions will aid in forecasting when and where consumptive versus predation‐risk effects should dominate.

## CONFLICT OF INTEREST

The authors declare no competing interests.

## AUTHOR CONTRIBUTION

**Jessica L. Pruett:** Conceptualization (equal); Data curation (lead); Formal analysis (lead); Investigation (lead); Methodology (equal); Visualization (lead); Writing‐original draft (lead); Writing‐review & editing (lead). **Marc J. Weissburg:** Conceptualization (equal); Funding acquisition (lead); Investigation (supporting); Methodology (equal); Validation (equal); Writing‐original draft (supporting); Writing‐review & editing (supporting).

## Data Availability

The data collect from this study are available in the Dryad Digital Repository: https://doi.org/10.5061/dryad.djh9w0vzb
